# Nystagmus‐related FRMD7 gene influences the maturation and complexities of neuronal processes in human neurons

**DOI:** 10.1002/brb3.1473

**Published:** 2019-11-19

**Authors:** Jiali Pu, Shaobing Dai, Ting Gao, Jing Hu, Yi Fang, Ran Zheng, Chongyao Jin, Baorong Zhang

**Affiliations:** ^1^ Department of Neurology Second Affiliated Hospital College of Medicine Zhejiang University Hangzhou China; ^2^ Department of Anesthesiology Women's Hospital School Of Medicine Zhejiang University Hangzhou China

**Keywords:** CRISPR/Cas9, FRMD7, idiopathic congenital nystagmus, induced neurons, neuronal outgrowth, Rho GTPases

## Abstract

**Aims:**

Idiopathic congenital nystagmus (ICN) is an oculomotor disorder caused by the defects in the ocular motor control regions of the brain. Mutations in FRMD7, a member of the FERM family of proteins, associated with cytoskeletal dynamics, are the most frequent causes of X‐linked ICN. Previous studies illustrated that FRMD7 is involved in the elongation of neurites during neuronal development; however, almost all the studies were performed on mice cell models. The complexity in the human neuronal network might suggest a unique vulnerability of human neurons to FRMD7 mutations.

**Methods:**

Herein, we successfully established human neuronal cell models with FRMD7 mutations, from fibroblasts‐reprogrammed neurons (iNs). In these neurons, the complexity of the neuronal processes was measured by the induced ratio, total neurite length, the number of terminals, and the number of maturation neurons.

**Results:**

The complexity of the neuronal processes was greatly reduced during various reprogramming stages in the presence of FRMD7 mutations. Consistently, the expression of the three main Rho GTPases was significantly increased by FRMD7 mutations. Interestingly, a slightly diverse phenotype is observed in different derived neurons.

**Conclusion:**

We established ideal human neuron models and confirmed that the mutation in FRMD7 influences the maturation and complexities of neuronal processes, which might be involved with the Rho GTPase signaling.

## Graphical abstract

This research established ideal human neuron models and confirmed that the mutation in FRMD7 influences the maturation and complexities of neuronal processes.

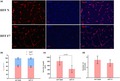

## 1 INTRODUCTION

The mutations in the FERM domain‐containing protein 7 (*FRMD7*; NM_194277) are the main causes of X‐linked form infantile nystagmus (IN; Choi et al., 2018; Tarpey et al., 2006; Wu et al., 2019). Previous studies illustrated that FRMD7 protein mediated the outgrowth of neurites in mouse neuroblastoma (Neuro‐2a) cells (Betts‐Henderson et al., 2010; Pu et al., 2012). In addition, in vivo studies on transgenic mice further verified that FRMD7 is a key regulator in forming asymmetry neuronal circuit (Yonehara et al., 2016). However, the mechanism of FRMD7 underlying the neuronal development and its involvement in X‐linked ICN pathogenesis remains unclear.

In the previous studies, we demonstrated that FRMD7 mutations altered the length of RA‐induced neurites in Neuro‐2a cells (Pu et al., 2012). In addition, the interaction between FRMD7 and Rho GTPases signaling (Pu et al., 2013) was identified in the non‐neuronal cell line. Moreover, Watkins et al. (2013) suggested that the interaction between FRMD7 and CASK promotes the membrane extension during neurite outgrowth; the study was also performed on mouse cell line. Hitherto, several studies based on mice‐derived neuron models confirmed the effects of FRMD7 on neuronal development. The complexity in human neuronal network might suggest a unique vulnerability of human neurons to FRMD7 mutations. Thus, the lack of an ideal human‐derived neuron model has limited the study of the pathogenesis of FRMD7 mutation.

Recently, induced neurons (iNs) reprogrammed from the human somatic cells offer a promising shortcut for obtaining specific neurons for in vitro modeling of neurological disorders (Ambasudhan et al., 2011). The direct conversion of human fibroblasts into mature and functional neurons relies on a couple of key transcription factors (TFs; Abdullah, Pollock, & Sun, 2012; Ambasudhan et al., 2011). Moreover, the development of programmable nucleases provides a powerful tool for modifying the target genome sequences. Especially, the clustered regularly interspaced short palindromic repeats (CRISPR)/Cas9, based on the introduction of DNA double‐strand breaks, facilitate the homologous recombination to target the endogenous genes in human somatic or iPSC cells effectively for reporter knockin, gene knockout, and gene correction (Cong et al., 2013; Pu, Frescas, Zhang, & Feng, 2015).

In the present study, we applied the CRISPRe/Cas9 gene editing tool to successfully generate random mutations (deletion, missense, or repetition mutations) into exon 2 of FRMD7 gene in two human fibroblasts cell lines MRC‐5 and HFF. Then, induced neurons were achieved from the direct conversion of fibroblasts (with or without FRMD7 gene mutations) via TFs and chemicals. Using these human‐derived neurons, we identified that the mutations of FRMD7 gene influence the efficiency of fibroblasts that reprogrammed the neurons from MRC‐5 cells, but not from HFF cells. Furthermore, FRMD7 mutation significantly reduced the length of total neurites but not the number of terminals; it decreased the ratio of mature neurons marked by Map2 from both the fibroblast‐induced neurons. The results showed that the FRMD7 mutation obviously decreased the complexities of neuron maturation. Furthermore, we found that the FRMD7 mutations modify the expression of Rho GTPases (Rho, Rac1, and Cdc42) in a time‐dependent manner. In summary, we established different types of human‐derived FRMD7 mutant neuron models and confirmed the effects of FRMD7 mutations on neuronal growth in human neurons for the first time. Also, the effect of FRMD7 mutation on GTP was explored.

## 2 MATERIALS AND METHODS

### 2.1 CRISPR/Cas9 vector construction and the activity assay

The human codon‐optimized Cas9 and chimeric guide RNA expression backbone plasmid pSpCas9 (BB)‐2A‐GFP (PX458) were obtained from Addgene (plasmid # 48138). A pair of annealed oligos that specifically targeted the exon 2 of human FRMD7 gene (*sgRNA* sequence: GATTTAGATGGCTGCAACTCAGG) had been cloned into the guide RNA. The oligos are designed according to the target site sequence (20 bp) and flanked on the 3′ end by a 3 bp NGG PAM sequence as previously described (Ran et al., 2013).

The surveyor nuclease assay was used to detect the activity of the FRMD7 CRISPR vector. Human embryonic kidney 293FT cells were transfected with plasmid DNA using Fugene 6 transfection reagent (Bio‐Rad). Cells were incubated at 37°C for 72 hr post‐transfection before genomic DNA extraction using the QuickExtract DNA extraction kit (Qiagen) according to the manufacturer's protocol.

The genomic region surrounding the FRMD7 CRISPR/Cas9 target site was amplified, and the PCR products were purified using QiaQuick Spin Column (Qiagen) following the manufacturer's protocol. An equivalent of 400 ng of the purified PCR products was mixed with 1.5 μl of 10× Taq polymerase PCR buffer (Invitrogen) and distilled water to a final volume of 15 μl. The heteroduplex was formed by a reannealing process: 95°C for 10 min, 95–20°C ramping at −0.3°C/s, and holding at 4°C. After reannealing, the products were treated with Surveyor nuclease and enhancer S (Transgenomics) following the manufacturer's recommended protocol and analyzed on 2% ethidium bromide agarose gel. The images were captured using a gel imaging system (Bio‐Rad), followed by the quantification was based on relative band intensities.

### 2.2 Materials

FUW‐tetO‐LoxP and pLKO.1/p53shRNA were purchased from Addgene. The GSK inhibitor CHIR 99021 was purchased from Reagents Direct (R&D). Dorsomorphin dihydrochloride and SB431542 were obtained from Tocris. Y27632 was purchased from Sigma‐Aldrich, and Purmorphamine was obtained from Swlleckchem. The Recombinant human factors (GDNF, BDNF, and NGF) were purchased from PeproTech. Human Ascl1 and miR‐124 sequences were subcloned to the *EcoRI* site in the FUW‐tetO‐LoxP vector (Jiang et al., 2015). All the plasmids were confirmed by sequencing directly.

### 2.3 Cell cultures and virus transfection

The human foreskin fibroblast cell line (HFF) and human fetal lung fibroblast MRC5 (Medical Research Council cell strain 5) were purchased from ATCC Global Bioresource Center and cultured in Dulbecco's modified Eagle's medium (DMEM; Invitrogen) containing 10% fetal bovine serum (FBS; Invitrogen), 10% penicillin, and 10% streptomycin. The cultures were maintained in 5% CO_2_ at 37°C and passaged every 2–3 days. Lentivirus production and fibroblasts infection were performed as described previously (Maherali et al., 2008). The titred of viruses was tested by the p24 levels ELISA kit from ZeptoMetrix Corporation. In the induced neuron experiment, fibroblasts were plated at a density of 5 × 10^3^ cm^−2^; after 24 hr, cells were infected with a couple of TFs combinations (Ascl1, miR‐124, and hp53shRNA each at MOI 10) in the presence of polybrene (8 μg/ml). After 16 hr transfection, the virus‐containing media was changes with induction medium: Dulbecco's modified Eagle's medium Nutrient Mixture F‐12 (DMEM/F‐12) supplemented with N2 (Invitrogen), B27 (Invitrogen), 20 µM vitamin C (Sigma), 1 µM Purmorphamine, 3 µM CHIR99021, 20 µM glial‐derived neurotrophic factor (GDNF), 20 µM brain‐derived neurotrophic factor (BDNF), 20 µM nerve growth factor (NGF), doxycycline (Dox, 1 μg/ml), and 10 µM Y27632. After 8 days induction, the culture medium was replaced with mature medium (induction medium without Purmorphamine, CHIR99021, and Dox). All media additives except BDNF, GDNF, and NGF were removed from the induction media from day 11.

### 2.4 Immunofluorescence and morphological analysis of neurites

Cells were seeded in 12‐well plates and fixed with 4% formaldehyde in PBS for 15 min at room temperature (RT) and blocked in 3% BSA in PBS for 60 min at RT, followed by incubation in primary antibody overnight at 4°C and secondary antibody for 2 hr at RT. The antibodies' sources, catalogue numbers, and dilutions are listed in Table S1. The morphological characters were quantified by a confocal laser scan microscope (Leica TCS SP5 X; Leica). Tuj1^+^, MAP2^+^, and DAPI^+^ cells were counted in a blinded manner from 10 randomly selected images (×10 magnification) for each condition. The length of the neurites was measured as previously described (Tegenge, Roloff, & Bicker, 2011). Briefly, 20–30 Tuj1^+^ neurons were traced and analyzed blindly for the total length of the neurites using Image J with the Neuron J. The number of terminals was counted via tracing. In order to avoid any ambiguity, neurons with overlapping neurites were excluded. The data presented are the mean of three independent experiments, shown as the mean ± standard error of the mean (*SEM*). For neurites length and number of terminals analysis, 250 cells per experiment were scored.

### 2.5 Estimation of Rho GTPases expression

Real‐time quantitative PCR (qRT‐PCR) was performed on total RNA isolated from induced neurons at different time points as described previously (Livak & Schmittgen, 2011). First‐strand complementary DNA (cDNA) was synthesized using SuperScript First‐Strand Synthesis System (Life Technologies, Invitrogen). For each sample, 5 μl cDNA product was used as the template for PCR amplification in a 25 μl reaction volume containing iQTM SYBR Green Supermix (Bio‐Rad) and 200 nM of each primer. The reaction was conducted using iCyler and iQ software (Bio‐Rad). The PCR conditions included an initial denaturation step of 4 min at 95°C, followed by 40 cycles of PCR consisting of 30 s at 95°C, 30 s at 60°C, and 30 s at 72°C. Each reaction was carried out in triplicate independently. The average threshold cycle (Ct) values from the triplicate PCR reactions for a gene of interest (GOI) were normalized against those for *GAPDH* from the same cDNA sample. The fold change of GOI transcript levels between sample *X* and sample *Y* is calculated as 2^−∆∆Ct^, where ∆Ct = Ct (GOI) − Ct (GAPDH), and ∆∆Ct = ∆Ct (*X*) − ∆Ct (*Y*) (Livak & Schmittgen, 2011).

### 2.6 Statistical analysis

All data are expressed as mean ± *SEM*. Unpaired, two‐tailed Student's *t* tests were performed using SPSS16.0 software (SPSS, Inc.) to evaluate whether the two groups were significantly different from each other. The sample size was selected based on the previous studies in the field with similar measurements. None of the sample was excluded from the current analysis. *p* < .05 was considered to be statistically significant.

## 3 RESULTS

### 3.1 Mutagenesis of FRMD7 in human fibroblast‐reprogrammed neurons

**Figure 1:**
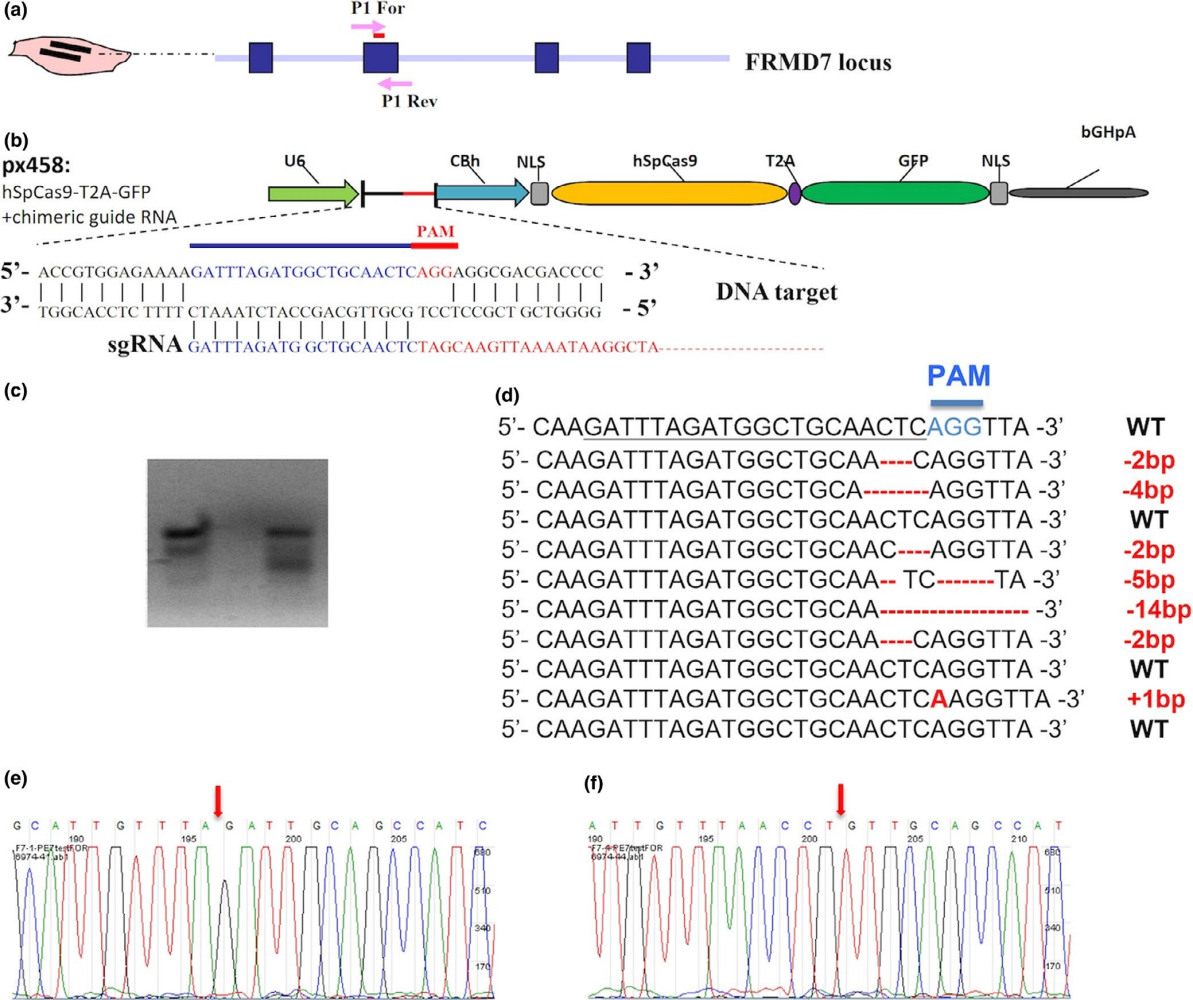
CRISPR/Cas9‐mediated mutagenesis of FRMD7 gene in human fibroblast cell line. (a, b) Schematic representation of the targeting strategy for the FRMD7 locus using CRISPR/Cas9. The PCR primer sites for homologous recombination test are indicted as arrows (P1 For and P1 Rev). The exons of the FRMD7 gene locus are indicated by blue boxes, and arrows indicate the genomic site cut by FRMD7‐CRISPR/Cas9. PX458: CRISPR/Cas9 template plasmid; sgRNA: guided RNA. (c) FRMD7‐CRISPR/Cas9 activity assay by site‐directed mutagenesis assay. FRMD7‐CRISPR/Cas9 was transiently transfected in human embryonic kidney 293FT cells. Prior to PCR amplification, genomic DNA was digested with *DdeI*, which was present at the FRMD7‐CRISPR/Cas9 target site. (d) The sequencing results of the clones. Out of 10, seven of the randomly sequenced clones harbored the mutations. (e, f) Examples of the mutated clones showing the mutant sites

**Figure 2:**
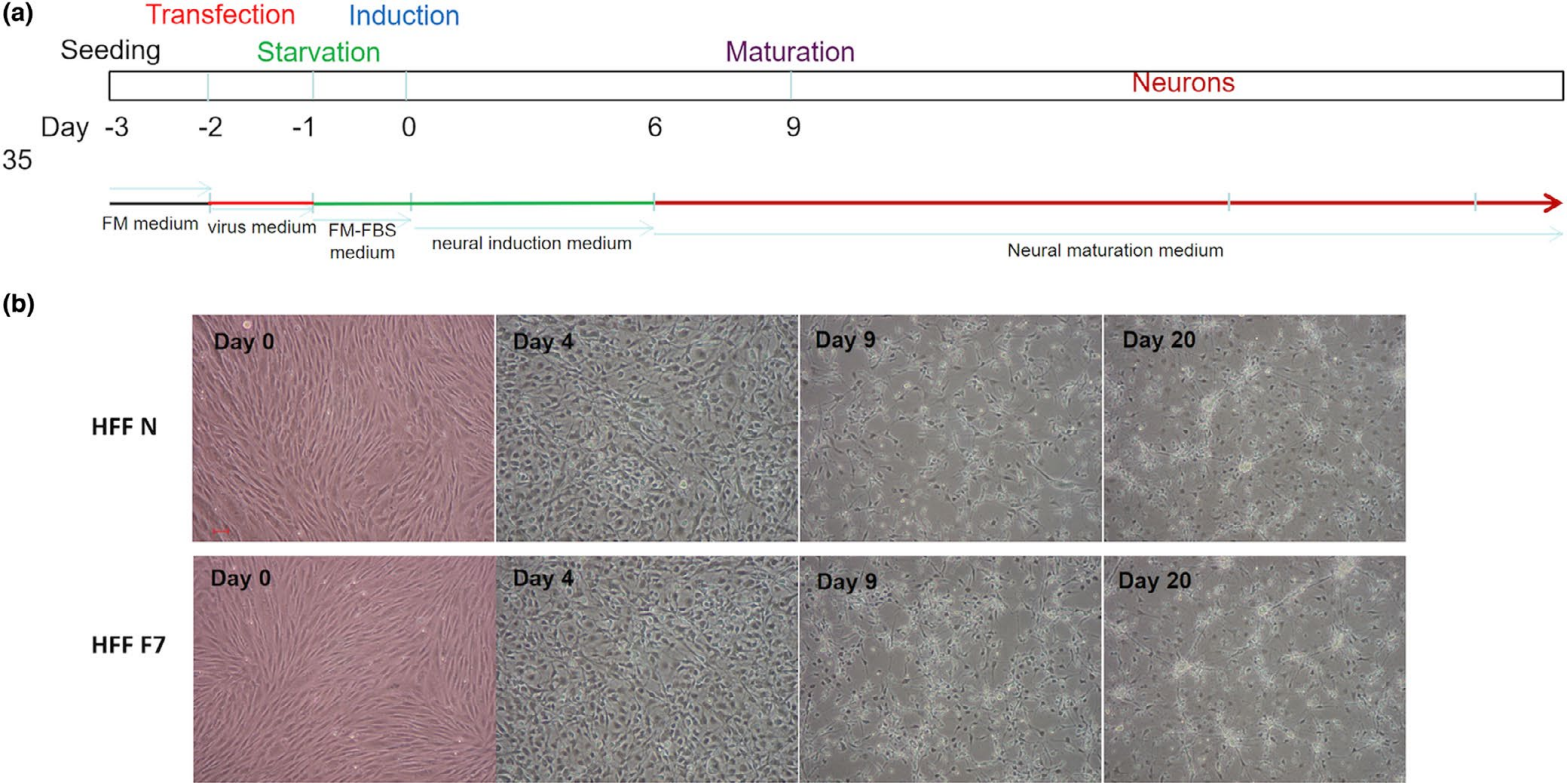
Direct conversion of human fibroblasts into neurons. (a) Conversion of fibroblast cells into neurons. Cells were reprogrammed to induced neurons (iNs) in neural induction medium with lentiviruses expressing Ascl1, p53 shRNA, plus miR124. FM: Full medium. (b) Cell morphology during HFF fibroblast reprogramming. Day 0: fibroblast cell morphology; day 4: the cells gradually changed their morphological characteristics; days 9 and 20: The sharp of the cells exhibited neuron‐like morphology. F7, FRMD7 mutant; N, control; scale bars: 100 μm

As described above, several studies have shown that FRMD7 influenced neuronal outgrowth in mouse cell line model; however, the evidence of its effect on human‐derived neuron is a limitation. In this study, we utilized the CRISPR/Cas9 technology to make mutagenesis of the FRMD7 gene in the two human fibroblast cell lines. The efficiency of the mutated FRMD7 gene in the MRC‐5 and HFF cells is approximately 70% (Figure 1). MRC‐5 and HFF widely used in several reprogramming experiments were infected with tetracycline‐inducible lentiviruses expressing human Ascl1, miRNA124, and a constitutively active lentivirus for p53 shRNA (Chanda et al., 2014; Cheng, Pastrana, Tavazoie, & Doetsch, 2009; Jiang et al., 2015). The cells were cultured in induction medium with the protocol described in Figure 2 and Figure S1. Neuron‐like long processes started to emerge after 4 days of induction. Then, the cells were stained at day 9 with antibodies against b3‐tubulin (Tuj1), an early‐stage neuronal marker.

### 3.2 FRMD7 modifies the complexities of induced neuron process

**Figure 3:**
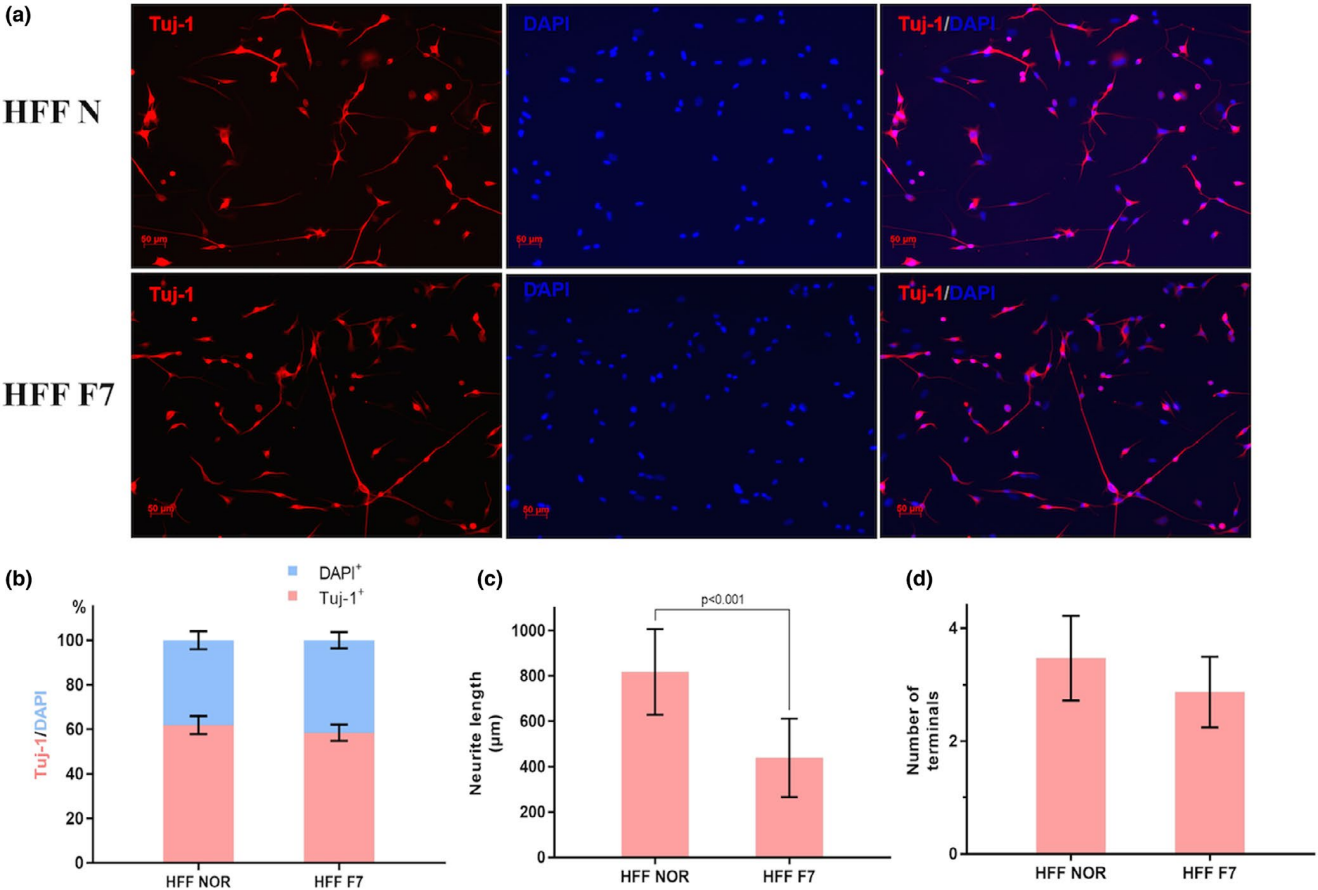
FRMD7 attenuation modifies the complexity of neurons from directly converted human fibroblasts. (a) The induced cells were stained at day 9 with antibodies against the neuronal marker b3‐tubulin (Tuj1). (b) Percentage of Tuj1^+^ cells in all cells (DAPI^+^) at day 9. *p < .05, unpaired, two‐tailed Student's *t* tests versus Tuj1^+^ in control, respectively. *n* = 6 wells from three independent experiments for each condition. Average numbers of Tuj1^+^ or DAPI^+^ cells per field under × 10 lenses were plotted. Total neurite length (c) and number of neurites (d) per Tuj‐1^+^ neurons. *p < .05, unpaired, two‐tailed Student's *t* tests versus control; *n* = 30–50 Tuj‐1^+^ neurons from three independent experiments for each condition

To verify whether the induced cells presented characteristics of early‐stage neurons and whether FRMD7 mutation influenced the reprogramming procedure, we stained with the neuronal marker Tuj‐1 at day 9. As a result, the mutations of FMRD7 in HFF did not influence the reprogramming efficiency (58.4 ± 6.3%) as compared to the control (61.9.5 ± 6.6%, *p* > .5). However, in the MRC‐5 cells, the result was different from that of HFF; the FRMD7 mutation significantly decreased the induced ratio of Tuj1/DAPI cells (67.5 ± 6.8%) as compared to the control (78.6 ± 9.1%). Using the Image J software, we traced the neurites and analyzed the total neurite length and the number of neurites for Tuj‐1^+^ cells derived from FRMD7 mutation. FRMD7 mutations were significantly decreased with respect to the total length of neurites (MRC‐5: 384.4 ± 33.4 vs. 642.6 ± 61.9, *p* < .05; HFF: 438.7 ± 44.7 vs. 817.7 ± 87.6, *p* < .001); however, the number of neurites is not affected (MRC‐5: 2.8 ± 0.1 vs. 3.2 ± 0.2, *p* > .05; HFF: 2.8 ± 0.3 vs. 3.4 ± 0.3, *p* > .05; Figure 3, Figure S2) as compared to the situation without mutagenesis. The data from MRC‐5 cells were illustrated in Figure S2.

### 3.3 FRMD7 influence induced the neuron maturation

**Figure 4:**
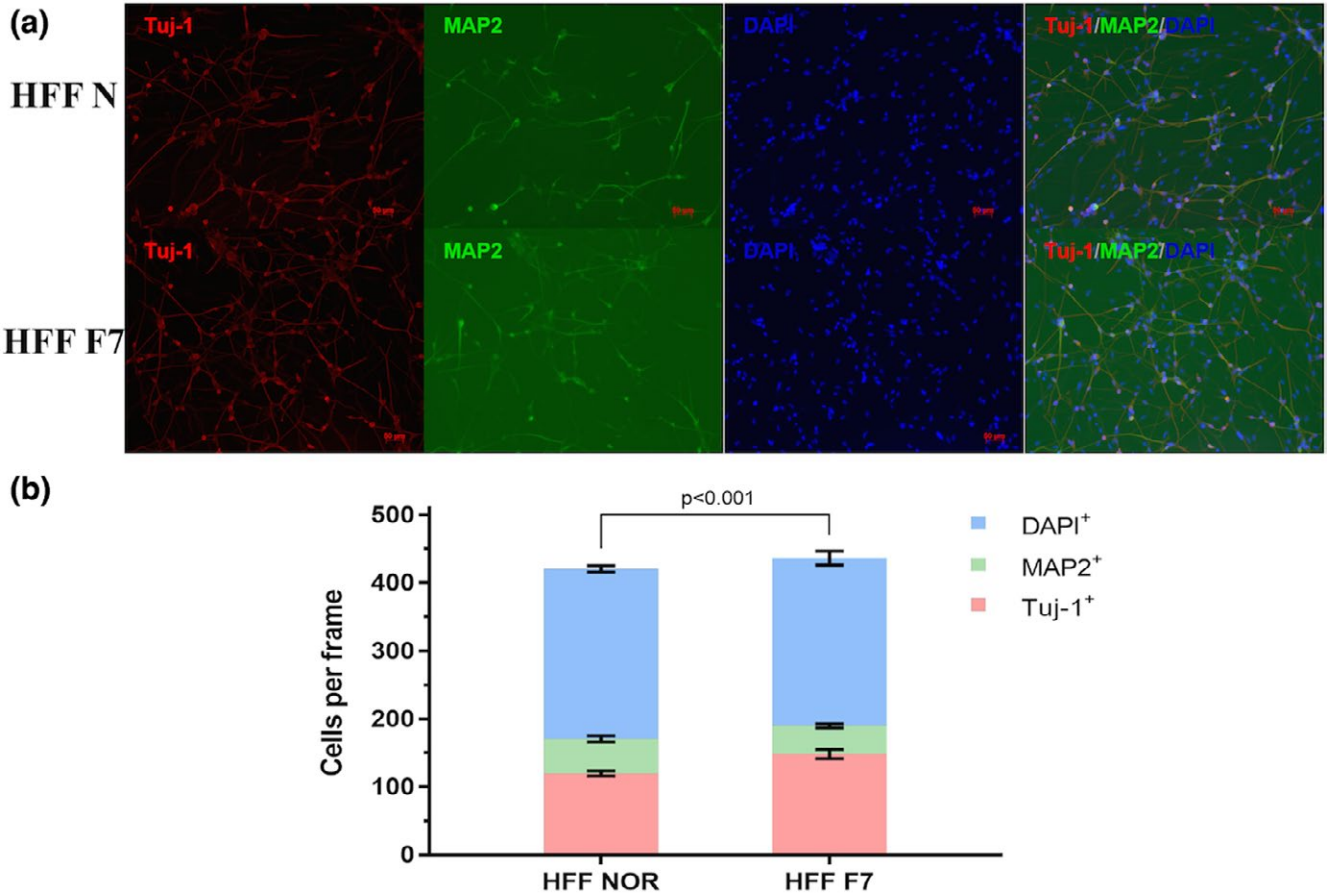
FRMD7 mutants influence the maturation of human fibroblasts‐reprogrammed neurons. (a) The induced cells were stained at day 15 with antibodies against the neuronal marker microtubule‐associated protein 2 (MAP2) in two different fibroblast cell lines. (b) Percentage of MAP2^+^ and Tuj1^+^ cells in all cells (DAPI^+^) at day 15. The ratio MAP2^+^/Tuj‐1^+^ was calculated. *p < .05, unpaired, two‐tailed Student's *t* tests versus MAP2^+^/Tuj‐1^+^ in control, respectively. The experiments were repeated three times, and the graphs represent the average of three independent experiments (columns, mean; bars, *SEM*.; *p < .05, **p < .01)

Furthermore, we investigated whether mutations of FRMD7 influence the maturation of these converted neurons. Immunofluorescence staining revealed the expression of neuron‐specific MAP2 and Tuj‐1 in the induced neurons at day 21 (Figure 4). The FRMD7 mutation significantly decreased the ratio of MAP2: Tuj‐1 cells (28.1 ± 4.1%) as compared to the control group (42.3 ± 5.2%). Initially, the protrusion process of MAP2^+^ mature neurons was analyzed; however, we found that these induced neurons showed significant aggregation in the later stages unable to measure the length of the neurites from single neuron.

**Figure 5:**
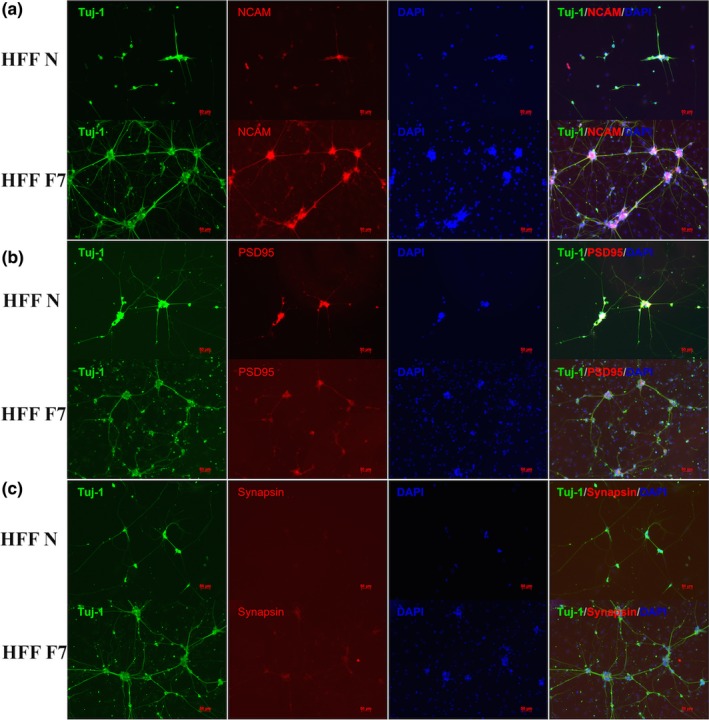
Characterization of the fibroblast‐induced neurons. Costaining of induced neurons with antibodies against Tuj‐1 and other neuronal markers, such as postsynaptic density‐95 (PSD95), synapsin, and neural cell adhesion molecule (NCAM). Scale bar, 10 mm

In addition, we detected other neuronal markers in late stage at day 32, such as NCAM, NeuN, and Synapsin (Figure 5). The induced neurons express most of these neuronal markers, which further confirm that these cells might comprise the optimal human‐derived neuronal model for disease research. The data from MRC‐5 cells were illustrated in Figure S3.

### 3.4 FRMD7 regulates the expression of Rho GTPases during induced time course

**Figure 6:**
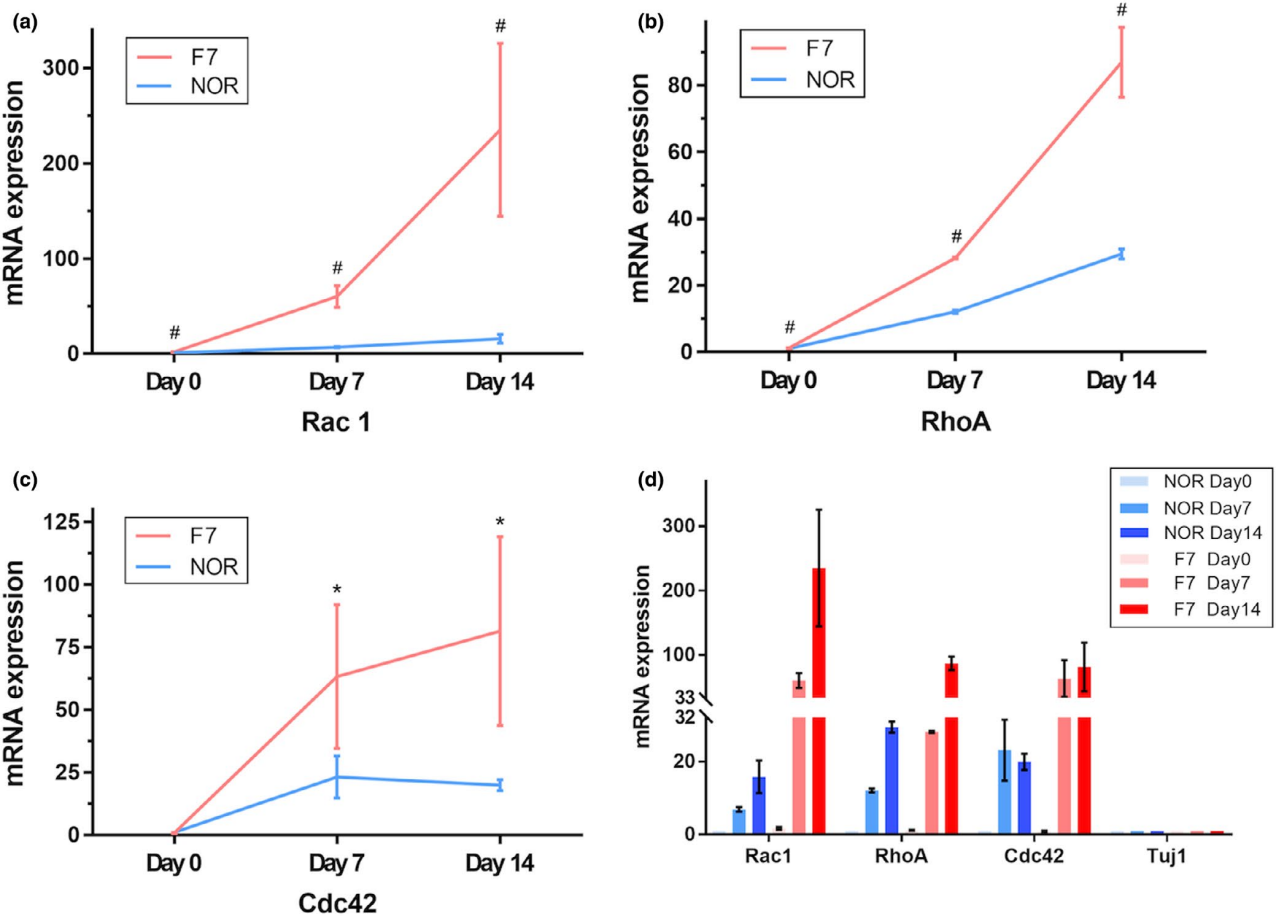
Mutations in FRMD7 increase the expression levels of neuron‐specific Rho GTPases during induced time lapse. The mRNA expression levels of three main Rho GTPases (*Rac1*, *Cdc42*, and *RhoA*) were measured by real‐time PCR during different time points. The expression of all of the three Rho GTPases was increased in the mutant FRMD7‐induced neurons and significantly for Rac1. With prolonged induction time, the increase in Rho GTPases expression was observed markedly. *p < .05, unpaired, two‐tailed Student's *t* tests versus control, respectively. Data are presented as the fold change in expression as compared to the controls. All experiments were performed in triplicate, and the graph shows the average for the three measurements (columns = means; bars = *SEM*; *p < .05, **p < .01 vs. controls)

The members of the Rho family of small G‐proteins (Rho GTPases) are key regulators of neuronal morphology (Jaffe & Hall, 2005; Luo, 2000). In a previous study, we had identified that FRMD7‐regulated neurite outgrowth was related to the Rho GTPases signal (Pu et al., 2013). In the present study, we detected the mRNA expression of Rho GTPases (*Rac1*, *RhoA*, and *Cdc42*) in the fibroblasts‐reprogrammed neurons with or without the FRMD7 mutations (Pu, Mao, Xu, Zheng, & Zhang, 2017). Next, a series of time‐lapse studies on mRNA expression profiles were conducted during different induced stages. Interestingly, we eventually obtained an inconsistent result with original study. The expression of Rho GTPases was increased while inducing differentiation; however, the FRMD7 mutation led to a sharper increase than the control (Figure 6). Nevertheless, the mechanism of differences in the expression file of Rho GTPases, which directly induced neurons, should be illustrated in future studies.

## 4 DISCUSSION

Using the CRISPR/Cas9 gene edit tool, the current study confirmed the mutagenesis of FRMD7 gene in human fibroblasts, which significantly influence the neurite outgrowth and neuronal maturation during cell differentiation into neurons. Although similar studies have been carried out previously, they are limited to nonhuman neuron models; some of them used the FRMD7 gene knock‐down via siRNA model or that established by transfection with mutant plasmids (Betts‐Henderson et al., 2010; Pu et al., 2012, 2017). Herein, FRMD7 mutation was established in iNs which mimics the cell model of congenital nystagmus caused by FRMD7 mutation, it made more favorable confirmation of the role for FRMD7 mutation on neuronal processes and maturation. This phenomenon laid the foundation for future research on pathogenesis. A recent study has shown that mouse and human fibroblasts can be directly reprogrammed into functional neurons (iNs) by skipping the pluripotent state with a cocktail of transcription factors (Ambasudhan et al., 2011; Jiang et al., 2015). Thus, the IN cells provide a novel and powerful system for studying molecular mechanism as an ideal neurological disease model. The present study, for the first time, discovered the role of the FRMD7 mutation in human fibroblast conversion neurons using Ascl1, miR124, and shRNA p53. However, in these three cell models, the effect of FRMD7 mutations on neuronal growth and differentiation is not identical, and the differences also prove that an ideal cellular model can simulate the occurrence of disease while investigating the underlying pathogenic mechanism.

In addition, the FRMD7 mutations modified the expression of Rho GTPases, in which RhoA, Rac1, and Cdc42 are best characterized in a time‐dependent manner, especially for Rac1. The Rho family of GTPases belongs to the Ras superfamily of guanine nucleotide‐binding proteins. As a result of the capability of modulating the dynamic changes and the rearrangement of cytoskeletons, the Rho GTPases have been highlighted as significant contributors for orchestrating the neuronal development (Jaffe & Hall, 2005; Luo, 2000). In the central nervous system development, the Rho GTPases switch between two states via regulators (GDIs, GAPs, and GEFs), a GTP‐bound active state, and a GDP‐bound inactive state. Moreover, the bidirectional regulation of Rho GTPases was essential for spatial and temporal signals to guide the downstream biological reactions, such as axon growth or retraction and synapse maturation or elimination, during the dynamics of neuronal morphology (Govek, Newey, & Van Aelst, 2005; Takano et al., 2017). Our previous studies have shown that human FRMD7 promotes the release of Rac1 from Rho GDIα, which in turn activates the Rac1 signaling pathway (Pu et al., 2013). In addition, FRMD7 regulates the expression of Rac1 and RhoA in stable SHSY‐5Y cells (Pu et al., 2017). In this study, we further demonstrated that mutant FRMD7 significantly influenced the expression of Rac1, Cdc42, and RhoA during the induction period of human fibroblasts‐reprogrammed neurons; nevertheless, the underlying mechanism is yet to be elucidated.

In conclusion, we established different types of human original FRMD7 mutant neuron models and confirmed the effects of FRMD7 mutations on the complexitiy of neurite growth and maturation in human neurons, as well as the effect of FRMD7 mutation on GTPase. Future studies would determine the FRMD7‐mediated regulation of the neuronal development and the correlation between FRMD7 and Rho GTPases or other signaling molecules. INs provide a novel and robust system for studying the cellular function, which might provide an in‐depth insight on the pathogenesis of ICN. Moreover, iN cells with FRMD7 mutations might seem to be an ideal disease model for future research.

## CONFLICT OF INTEREST

The authors have declared that no competing interests exist.

## Data Availability

The data that support the findings of this study are available from the corresponding author upon reasonable request.
