# Supplementary figures and images for: Nystagmus‐related FRMD7 gene influences the maturation and complexities of neuronal processes in human neurons
Source: Brain Behav. 2019 Nov 19;9(12):e01473. doi: 10.1002/brb3.1473 (PMC6908866; doi:10.1002/brb3.1473)

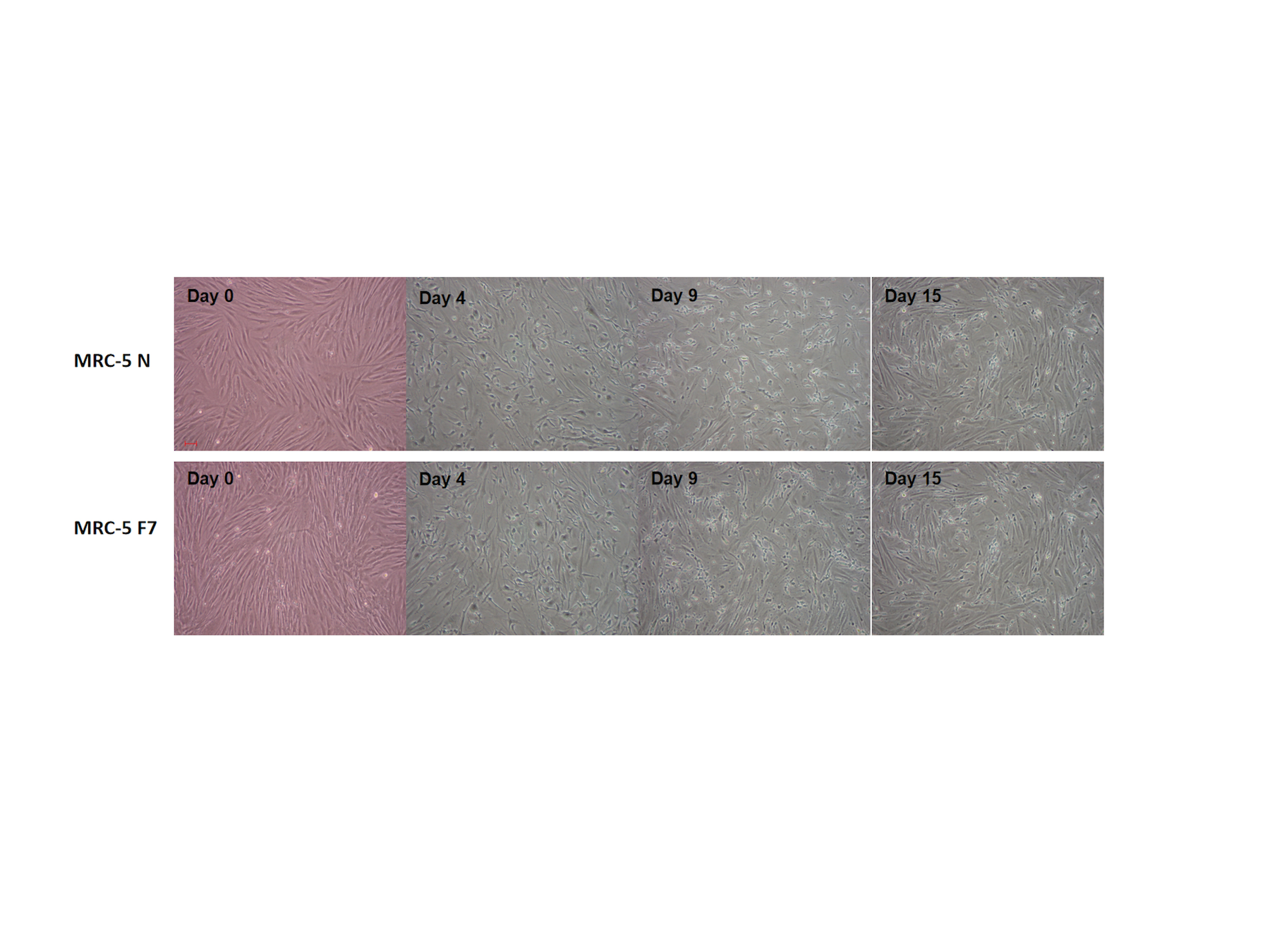

Supplement: Supplementary file 1 [file BRB3-9-e01473-s001.tiff]

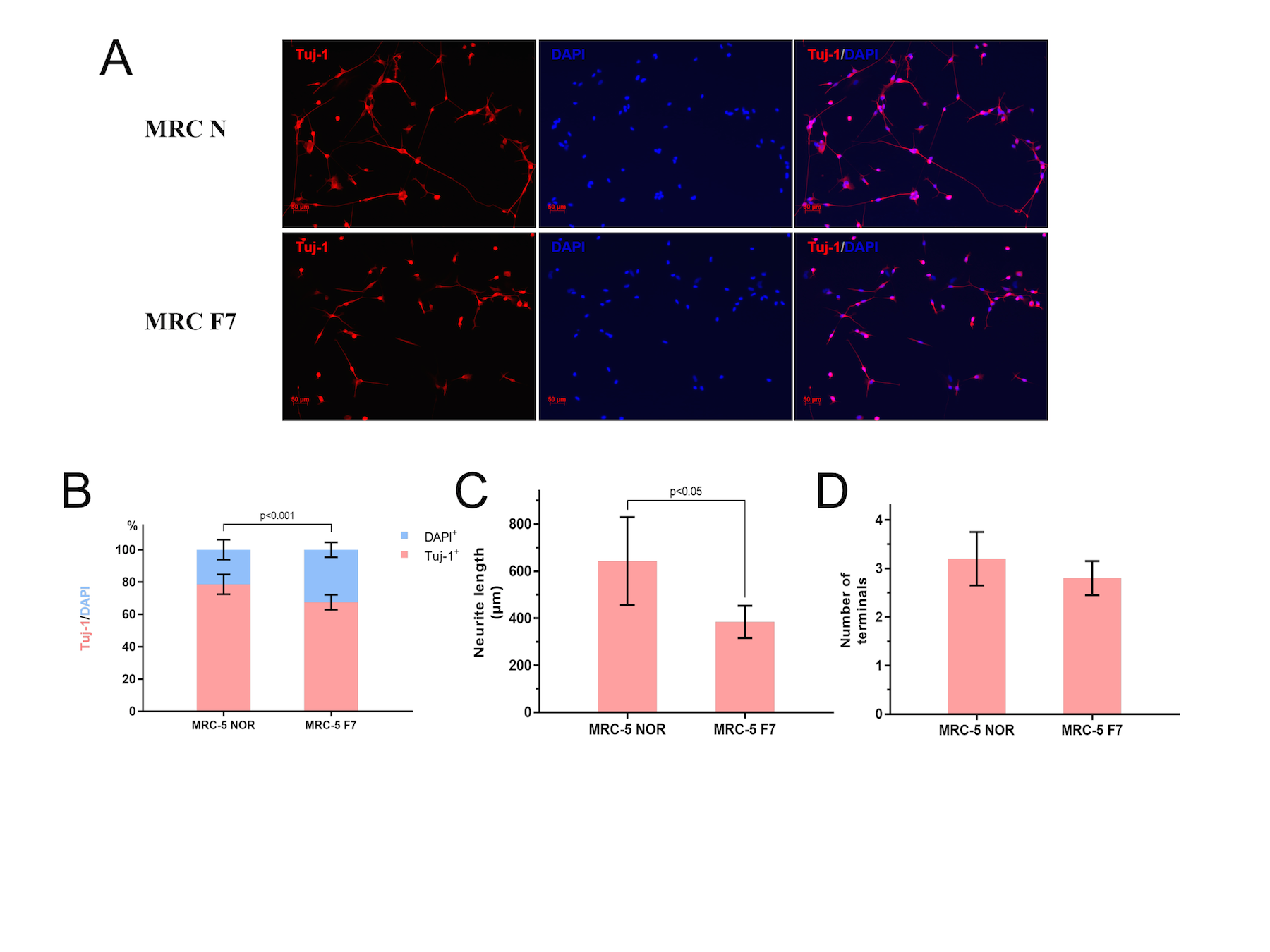

Supplement: Supplementary file 2 [file BRB3-9-e01473-s002.tiff]

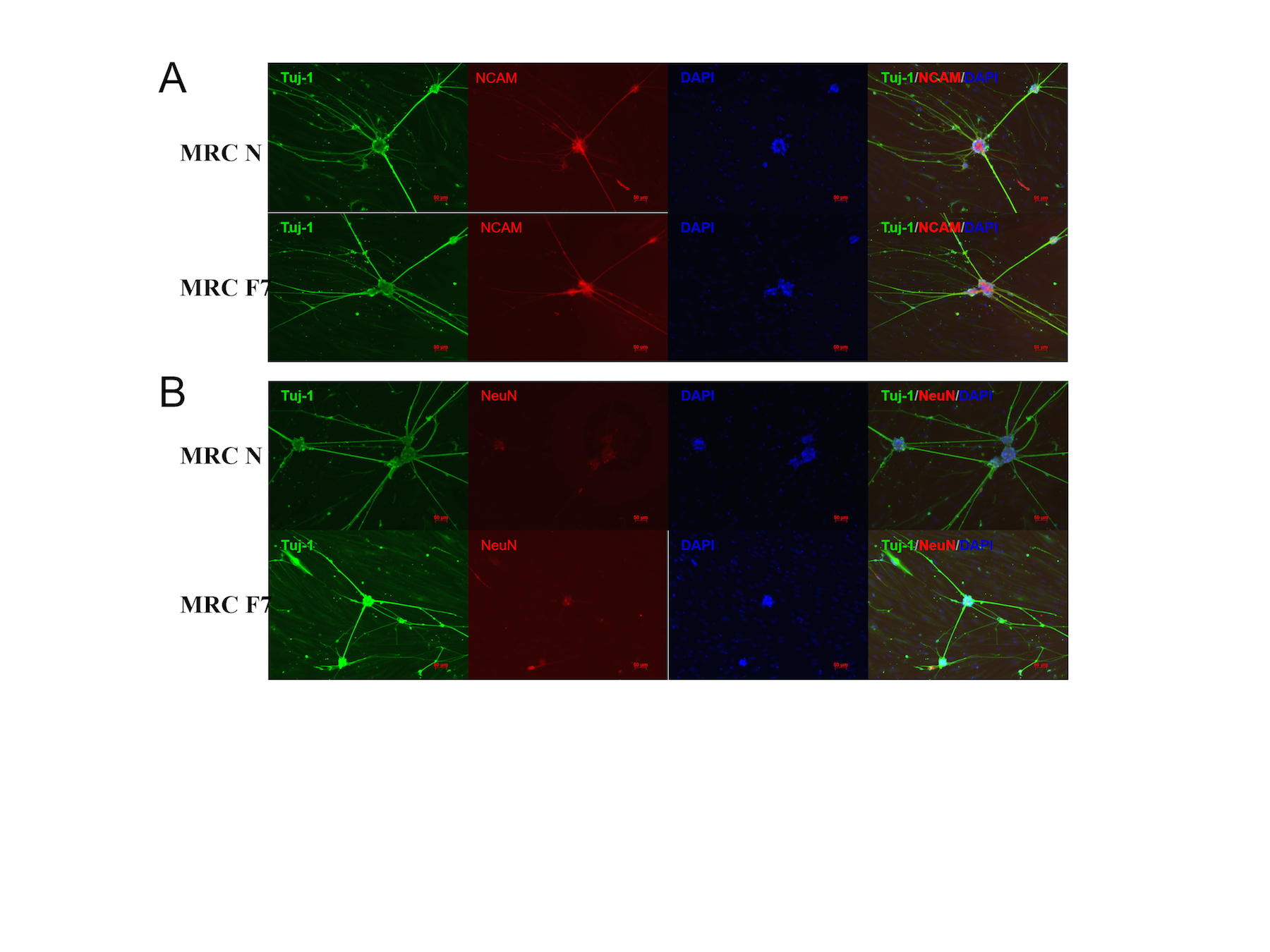

Supplement: Supplementary file 3 [file BRB3-9-e01473-s003.tiff]
