# Supplementary material for: Nystagmus‐related FRMD7 gene influences the maturation and complexities of neuronal processes in human neurons
Source: Brain Behav. 2019 Nov 19;9(12):e01473. doi: 10.1002/brb3.1473 (PMC6908866; doi:10.1002/brb3.1473)
Supplement: Supplementary file 4 [file BRB3-9-e01473-s004.docx]

# Supplementary Table 1: Antibodies and their Dilutions

| **Protein** | **Species of Antibody** | **Vendor (catalog #)** | **Dilution** |
| --- | --- | --- | --- |
| **Tuj-1** | Mouse | Covance (MMS-435P-250) | 1:1000 |
| **MAP2** | Mouse | Santa Cruz(sc-74421) | 1:1000 |
| **NeuN** | Mouse | Millipore (MAB377) | 1:500 |
| **PSD95** | Rabbit | Cell Signaling (2507s) | 1:1000 |
| **vGlut2** | mouse | Santa Cruz (SC-6260) | 1:250 |
| **NCAM** | mouse | Santa Cruz (SC-7326) | 1:500 |
| **synaptophysin** | rabbit | Thermo Scientific (PA1-1043) | 1:1000 |
| **AlexaFluor 488** | anti-Rabbit IgG | Life Technologies (A11008) | 1:2000 |
| **AlexaFluor 594** | anti-Rabbit IgG | Life Technologies (A11012) | 1:2000 |
| **AlexaFluor 488** | anti-mouse IgG | Life Technologies (A21202) | 1:2000 |
| **AlexaFluor 594** | anti-mouse IgG | Life Technologies (A11032) | 1:2000 |
| **AlexaFluor 594** | anti-mouse IgG2a | Life Technologies (A21135) | 1:2000 |
